# Crows Rival Monkeys in Cognitive Capacity

**DOI:** 10.1038/s41598-017-09400-0

**Published:** 2017-08-18

**Authors:** Dmitry Balakhonov, Jonas Rose

**Affiliations:** Dept. of Psychology, Ruhr-University Bochum, 44801 Bochum, Germany

## Abstract

The present study compares the ‘bandwidth of cognition’ between crows and primates. Working memory is the ability to maintain and manipulate information over short periods of time – a core component of cognition. The capacity of working memory is tightly limited, in humans correlated with individual intelligence and commonly used synonymously with cognitive capacity. Crows have remarkable cognitive skills and while birds and mammals share neural principles of working memory, its capacity has not been tested in crows. Here we report the performance of two carrion crows on a working memory paradigm adapted from a recent experiment in rhesus monkeys. Capacity of crows is remarkably similar to monkeys and estimated at about four items. In both species, the visual hemifields show largely independent capacity. These results show that crows, like primates evolved a high-capacity working memory that reflects the result of convergent evolution of higher cognitive abilities in both species.

## Introduction

A wealth of recent studies demonstrates a remarkable cognitive repertoire in corvid birds^1^. For instance, corvids spontaneously show analogical reasoning^2^, episodic-like memory^3, 4^, tool use^5–8^, complex social interaction^9–11^ and insight into the mental state of conspecifics^12^. These abilities surpass most mammals that have been tested in such paradigms and are on par with primates^1^. However, it is not yet definitive if crows evolved a general and flexible neural system for higher cognition, “specialized functions that acquire information about goals and means to select and coordinate among innate and well-established routines”^13^. This concept of higher cognition implies a domain-general system that can solve novel problems requiring ‘high mental effort’ - online information processing that goes beyond the retrieval of previously acquired stimulus-response associations^14^. Many of the abilities reported in crows could reflect domain specific adaptations. This criticism was raised because different corvid species were tested on specialized behaviors, such as tool use in new Caledonian crows or food caching in scrub jays with relatively few studies investigating abstract cognitive abilities^15^. One reason for this is the lack of a behavioral test for general cognitive aptitude (comparable to the intelligence quotient in humans) that can be applied across species. To construct a concise image of the cognitive abilities of a species we have to combine multiple tests and approaches including behavior, neurophysiology, ecology and evolutionary biology. The latter provide a strong indication that birds and mammals independently evolved neural structures for higher cognition^13, 16, 17^. Many corvid species are adapted to complex, variable environments. Here they demonstrate ecological flexibility along with complex foraging strategies and social behavior, driving factors to developing higher cognitive abilities rather than adapting to a specific ecological niche^18^. As an example, corvids are able to learn a basic ‘same-different’ concept, outperforming monkeys in an identical task; such abilities range beyond specific environmental demands for the species and require a flexible cognitive approach^19^. Strong evidence for the evolutionary convergence of higher cognition can be found at the neural level. Birds and mammals both evolved a large pallium (Latin for mantle) of the same relative size, internal connectivity and comparable functionality. In mammals, the pallium (mostly) evolved into the layered structure of the cortex while in birds it follows a nuclear organization without clearly visible layering^17, 20, 21^. This paradoxical combination of similarities and differences of its neural substrate supports the notion of a parallel evolution of cognition.

In primates including humans one of the core components of higher cognition is working memory (WM), the neural system for short-term storage and manipulation of information^22^. Many higher cognitive abilities, for instance planning and cognitive flexibility, are critically dependent on WM^23^ and the capacity of WM is closely correlated with individual fluid intelligence^24, 25^. There is a long standing debate on many aspects of WM-capacity be it the exact number of items that can be stored, task- and domain-specificity, the mechanisms of chunking information in WM (grouping of stimuli such that they can be stored in integrated representations thereby reducing WM-load) or appropriate analyses to assess WM-capacity (see Cowan^26^ for overview and comments). To date there is wide consensus that healthy humans have a capacity of around four items^26, 27^, the development of this capacity continues until adolescence^28^. Recent electrophysiological work in humans and monkeys lends strong evidence to the idea that the two cerebral hemispheres have an independent capacity^29–32^. The neural basis of WM is understood reasonably well and we know that the principal mechanism, sustained neural activation, is comparable between birds and mammals^33–38^. Equally comparable are the functional connectivity and neuropharmacology of the WM system^39^. Therefore, it is very likely that birds, like mammals, evolved WM as a general neural system for higher cognition^39^. In fact, crows and primates use WM to briefly store abstract rules that guide behavior^40–43^ and to maintain multimodal information^44, 45^.

Here we compare the capacity of WM between crows and primates^29, 46^. We can interpret the presence of high capacity WM as indicator of overall cognitive capacity of a species. Additionally, if crows and primates independently evolved a high capacity WM this could imply that there are no alternative neural solutions for higher cognition.

## Results

**Figure 1:**
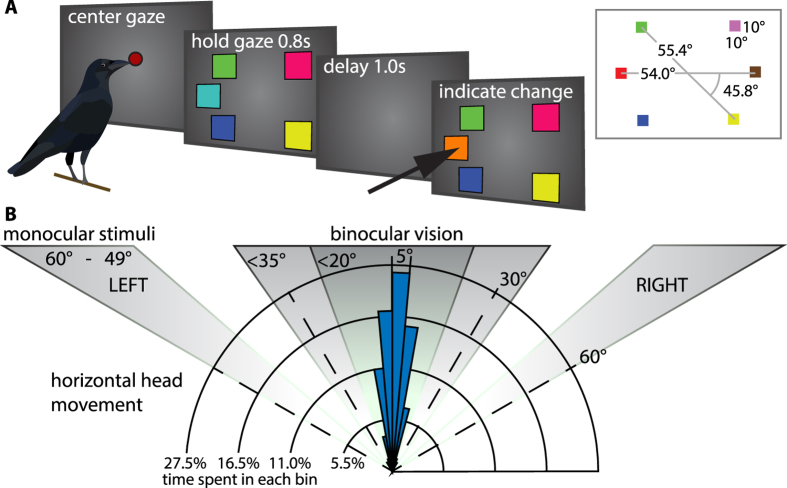
Behavioral protocol and gaze direction. **(A)** Behavioral protocol. After initiating a trial by placing the head in front of a red dot in the center of the screen, crows were presented with a stimulus-array of colored squares. Following a short memory delay during which the stimuli were not present, the animals viewed the choice array, i.e. the stimulus-array with one color exchanged. A single peck to the changed stimulus (indicated by arrow) resulted in reinforcement. Panel inset illustrates stimulus-placement and dimensions on screen (unit: degree visual angle). **(B)** Gaze direction and stimulus arrangement. Blue histogram indicates horizontal head-rotation (time spent in bins of 5 degree visual angle) of both animals during the entire experiment. Central grey field indicates binocular visual field of crows, light shading takes head-rotation into account. Lateral grey regions (left and right) indicate area of stimulus presentation on the monitor indicating that stimuli were only viewed with either the left or the right eye.

Two naïve crows (*Corvus corone*) were trained on a change localization paradigm that was closely modeled after a paradigm recently used to assess WM-capacity in primates (M*accaca mulatta* and *Maccaca fascicularis*)^29^ (Fig. 1A). The crows were trained for about ten months (roughly comparable to primates), after which they were tested for ten days, the results of this testing period are reported here. In the current experiment we utilized a head-tracking system to control the gaze-direction of the animals. Taking advantage of the comparatively small binocular visual field and by training the animals to hold the head centered and straight during stimulus presentation, we controlled which eye viewed the stimuli (Fig. 1B).

**Figure 2:**
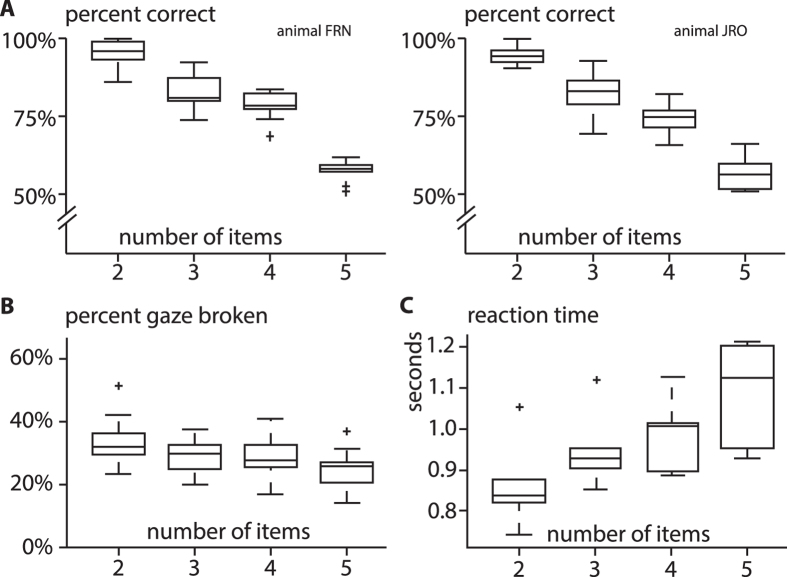
Crows’ performance is modulated by number of items in WM. All box-plots display median, 25^th^ and 75^th^ percentile, whiskers display the full extent of the data, crosses indicate outliers. **(A)** Performance of each animal on attempted trials as a function of number of items in stimulus-array (mean performance animal FRN 2: 94%, 3: 82%, 4: 74%, 5: 56%; animal JRO 2: 95%, 3: 82%, 4: 78%, 5: 57%). **(B)** Percent gaze-breaks of both animals on started trials as a function of number of items in stimulus-array (mean 2: 34%, 3: 29%, 4: 29%, 5: 24%**)**. **(C)** Reaction time of both animals on correct trials as a function of number of items in stimulus-array (mean 2: 0.86 s, 3: 0.95 s, 4: 0.99 s, 5: 1.09 s).

Both animals performed the paradigm on average 681 trials daily and overall performance was well above chance for each WM-load (2, 3, 4 and 5 items). With an increasing number of items in the stimulus-array, however, performance decreased significantly (Fig. 2A, ANOVA both animals: F(1,79) = 461.36, p < 0.01, η_p_^2^ = 0.855; animal FRN: F(1,39) = 211.35, p < 0.01, η_p_^2^ = 0.848; animal JRO: F(1,39) = 250.18, p < 0.01, η_p_^2^ = 0.868). This performance-modulation cannot be explained by the animals ‘opting-out’ of more difficult trials since gaze-breaks decreased slightly but significantly with increasing load on WM (Fig. 2C, Supplementary Fig. S2, ANOVA both animals: F(1,79) = 24.36, p < 0.05, η_p_^2^ = 0.238; animal FRN: F(1,39) = 15.77, p < 0.01, η_p_^2^ = 0.293; animal JRO: F(1,39) = 9.07, p < 0.01, η_p_^2^ = 0.193). Along with the decreased performance, the reaction times increased with higher WM-load suggesting higher cognitive demand (Fig. 2C, ANOVA both animals: F(1,23) = 16.45, p < 0.01, η_p_^2^ = 0.428; animal FRN: F(1,23) = 17.31, p < 0.01, η_p_^2^ = 0.440; animal JRO: F(1,23) = 8.7, p < 0.01, η_p_^2^ = 0.283). Reaction times were also increased on incorrect compared to correct choices (hit: 984 ± 5 ms; false alarm 1678 ± 13 ms; mean ± sem; ANOVA both animals: F(1,9175) = 4220.9, p < 0.01, η_p_^2^ = 0.315; animal FRN: F(1,4370) = 1766.57, p < 0.01, η_p_^2^ = 0.288; animal JRO: F(1,4804) = 2481.65, p < 0.01, η_p_^2^ = 0.340). This could indicate additional cognitive operations when the animals were uncertain – for example in the form of additional scanning of the choice-display.

**Figure 3:**
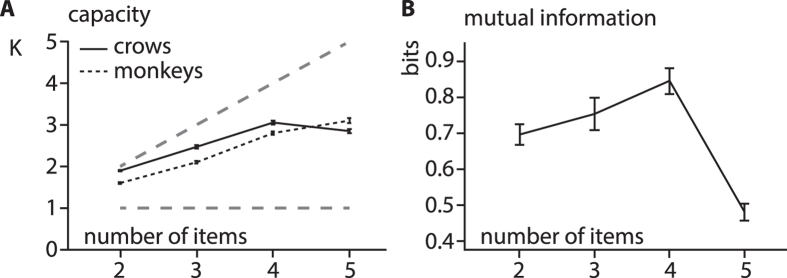
Capacity of WM is comparable between crows and primates. **(A)** Capacity K as a function of number of items in stimulus-array. Solid black line depicts capacity of both crows, dotted black line depicts capacity of both monkeys (data published in^46^). Grey dashed lines depict chance-level and optimal performance. Error-bars are SEM. **(B)** Mutual information of both crows as a function of number of items in stimulus-array. Information about stimulus-array increases until capacity is exceeded (4–5 items) at which point there is a loss of information. Error-bars are SEM.

For a direct comparison of the WM-capacity between crows and primates^29, 46^ we calculated capacity K (see methods) at each WM-load (Fig. 3A). We found that capacity K grows linearly up to four items at which point the crows show a very clear deterioration of performance. At a load of four items capacity K is 3.1 ± 0.05 (mean ± sem) for crows, which is well within the range reported for primates^46^ and healthy humans^47^. We further calculated mutual information to quantify stimulus information available to the animals at each WM-load (Fig. 3B). The underlying idea of this analysis is that representing a larger number of items requires more information than a smaller number, information that can be estimated from performance. We found that stimulus information increases with increasing number of items until it drops between four and five items. We further estimated capacity from behavior using a modelling approach^29^ resulting in an estimate of 3.36. Given the slightly different structure of the results between crows and primates (crows performed higher at low load and lower above capacity, Fig. 3B), this approach resulted in an unsatisfactory fit for the data (R2 = 0.79, linear model for comparison: R2 = 0.86), a very high estimate for baseline (88.6%) and consequently low estimate for capacity.

**Figure 4:**
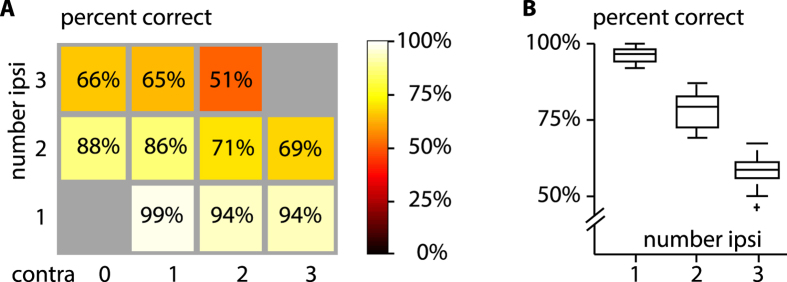
Capacity of working memory is independent between the two hemispheres. **(A)** Performance of both animals as a function of number of stimuli on target side (number ipsi) and number of items contralateral to the target (contra). Background shading indicates mean percent correct on all attempted trials. **(B)** Performance of both animals as a function of items on the target side **(**mean, 1: 96%, 2: 78%, 3: 58%). Box-plots display median, 25^th^ and 75^th^ percentile, whiskers display the full extent of the data, crosses indicate outliers.

We found that in crows, much like in monkeys, performance is strongly modulated by the number of items that are presented on the target side. While the number stimuli presented on the contralateral side has virtually no effect in the primate study it does affect the crows’ performance if at least one distracter is presented on the target side (Fig. 4). A two-way ANOVA calculated for both animals showed a significant main effect for number of items ipsilateral to the target (F(1,199) = 137.1, p < 0.01, η_p_^2^ = 0.412) but not number of items contralateral (F(1,199) = 1.27, p = 0.26, η_p_^2^ = 0.006) the interaction between both factors is small with a small effect size but is also significant (F(1,199) = 6.47, p = 0.012, η_p_^2^ = 0.032). Calculated for animals FRN and JRO individually, it showed a significant main effect for number of items ipsilateral to the target (animal FRN: F(1,299) = 214.1, p < 0.01, η_p_^2^ = 0.420; animal JRO: F(1,399) = 277, p < 0.01, η_p_^2^ = 0.412) but not number of items contralateral (animal FRN: F(1,299) = 1.56, p = 0.21, η_p_^2^ = 0.005; animal JRO: F(1,399) = 3.02, p = 0.11, η_p_^2^ = 0.006) the interaction between both factors is also significant for animal FRN (F(1,299) = 9.77, p < 0.01, η_p_^2^ = 0.032) and for animal JRO (F(1,399) = 13.07, p < 0.01, η_p_^2^ = 0.032). At the level of individual animals, the number of items ipsilateral to the target has a large effect on performance, the interaction between number of items ipsilateral and contralateral only has a small effect. For better compatibility with the previous monkey study^29^ we did an additional two-way ANOVA in a way identical to the study. The ANOVA showed a similar picture: a significant main effect for number of items ipsilateral to the target (F(1,9127) = 270.7, p < 0.01, η_p_^2^ = 0.029), but not number of items contralateral (F(1,9172) = 1.19, p = 0.28, η_p_^2^ = 0.0001) and small, but significant interaction between both factors (F(1,9127) = 15.7, p < 0.01, η_p_^2^ = 0.002).

The effect between the number of items ipsilateral and contralateral to the target was also tested using a general linear model with logistic regression (GLM). Here both factors (number of items ipsilateral and contralateral) had a significant influence on the model but the influence of the number ipsilateral was over three times that of the number contralateral (ipsilateral β = −1.41, p < 0.01, t = −34.20; contralateral: β = −0.42, p < 0.01, t = −13.81). We found no significant difference in performance between the left and right side (ANOVA animal FRN: F(1,4370) = 0.01, p = 0.929, η_p_^2^ < 0.0001; animal JRO: F(1,4804) = 0.11, p = 0.74, η_p_^2^ < 0.0001, performance at each stimulus location and side is displayed in Supplementary Fig. S1).

## Discussion

Here we report the working memory capacity of two carrion crows in a working memory paradigm that is virtually identical to a paradigm recently used with primates^29, 46^. Overall, the birds and the monkeys show a remarkably similar capacity of about four items. Compared to the monkeys, the crows show slightly higher performance below capacity with a steeper decline once their capacity is exceeded. Additionally, in both species capacity is largely dependent on the number of stimuli that are presented on the same side as the target. The number of items presented contralateral to the target have virtually no effect in monkeys but some effect in crows that is evident only when at least one distracter is presented on the target side.

We chose to investigate WM-capacity in crows because these animals have an impressive cognitive repertoire and, in mammals, WM is believed to be at the core of our flexible neural system for higher cognition. Importantly, the capacity of WM is often viewed as an indicator of overall cognitive capacity. This notion, however, is not accepted by the entire field^26^. Some researchers postulate that the observed limitations reflect mere limitations of the underlying sensory system but not a separate bottleneck in WM. Additionally the precise estimate of WM-capacity can be highly dependent on specific task-demands or analyses. Therefore, we carefully adapted a paradigm that was recently used with primates for the work with crows. This comparative approach is possible because birds, like primates have an excellent visual system with high accuracy and color resolution^48^. We used identical stimulus-composition, task-structure and durations, stimulus randomization and analysis techniques. The most notable difference between the paradigms was that the monkeys were trained using eye movements and were head-restrained, the crows on the other hand were head-free and responded with pecks on a touchscreen monitor. However, both species were required to fixate their gaze on the center of the screen during stimulus presentation. Given this high comparability between the paradigms it seems unlikely that our results reflect major technical differences that warrant a different interpretation of the data.

Recent comparative work has estimated WM-capacity in monkeys^29, 46, 49^ and even compared monkeys and pigeons^50–52^. Among these studies, rhesus macaques were estimated to have a capacity of about two^51, 52^ or four^29, 46^. Capacity of pigeons was estimated at about one^51, 52^ or two^50^. While monkeys clearly exceeded the pigeons in a direct comparison with identical protocol and analysis, the vastly different estimates between studies highlight the difficulties when different paradigms or analyses are used.

In addition to the overall capacity we also examined in how far the allocation of capacity is comparable between crows and primates. Buschman *et al*.^29^ reported that monkeys have independent capacities in both visual hemifields – only the number of stimuli presented on the same side as the target affected performance, the number of stimuli contralateral to the target had virtually no effect on performance. Similarly, in crows the number of stimuli presented contralateral to the target had no effect on performance if only one item was presented on the target side. However, if multiple items were presented on the target side crows showed some small decrease in performance. This weak effect of the number of items presented contralaterally might reflect either a cognitive difference between the species or a technical difference. The former would suggest that crows show a stronger integration of information between the two cerebral hemispheres than monkeys. While possible, this seems somewhat counterintuitive given the complete crossing of the optic nerve and absence of a corpus callosum in the avian brain^48^. The latter, technical differences, might arise between the uses of eye-tracking in head-restrained primates that allowed for fast (240 Hz), near perfect control of visual stimulation. In the present study in crows only head position and orientation was controlled at 50 Hz (smoothed over 2 frames). While unlikely, it is therefore possible that, in crows, there was some contamination between the visual hemifields, for example through very brief eye/head-movements. Another difference may arise from the response type. Monkeys indicated their choice with a direct saccade from the central fixation target to the chosen color. Therefore they were unable to scan the choice stimulus-display with eye movements. The crows, on the other hand indicated their choice with a peck to the chosen color. Therefore, head-tracking was not continued into the choice period and the animals were able to briefly scan the stimuli before committing to a choice. Lateralized stimulus presentation during the choice period may be a relevant factor here. If, in this paradigm, capacity of WM is limited by the comparison between the memoranda and the choice stimuli then the ability to freely scan the choice stimuli may affect the degree of hemisphere-independence. All in all it seems unlikely that the very small effect of number of items contralateral to the target reflects a true species differences between crows and primate small technical differences between the studies seem a more likely explanation. Another behavioral similarity between crows and monkeys was the absence of hemispheric dominance, in both species overall performance was indistinguishable between items presented on the left and right side.

The most notable difference between crows and primates was in the pattern of performance with an increasing number of items. While the crows performed at higher levels below capacity, their performance showed a sharper decrease above capacity. This is most obvious in mutual information about the target display, where the monkeys show a plateau at capacity and the crows a clear drop in information. With only the behavioral data at hand this finding is difficult to explain, it could reflect a change in motivation or a mechanistic difference in WM between the species. This sharp decrease in the crows’ performance above capacity could also be explained by ‘continuous resource’ models that assume that each stimulus added into WM degrades the quality of the neural representation of all stored stimuli^53^. Neural recordings in primates showed that, within one hemifield, the loss of neural information is consistent with continuous resource models^29^. Therefore, this interpretation is consistent with our data but cannot explain the observed difference between crows and primates. A detailed analysis of the neural processes underlying the capacity limitations of WM in crows will be necessary to better understand differences and similarities between the species. Single cell recordings have already been performed in primates in this paradigm and taking a similar approach in crows will help to compare and understand the neural basis of WM capacity. At this point it is not possible to conclude if the mechanisms limiting the capacity of crows and monkeys at about four items are comparable or if the different pattern of performance reflects true mechanistic differences of WM.

This work adds to the growing evidence on corvid cognition, expanding our knowledge in comparatively young field that is still met with skepticism^15, 54, 55^. Most importantly, however, we demonstrate that corvids, like primates, evolved a neural system that can store and process multiple pieces of information simultaneously. This neural capacity for abstract and complex computation is comparable between the species, thereby enabling crows to perform cognitive processes like analogical reasoning and planning, potentially at the same level as rhesus macaques. Furthermore, if primates and corvids independently evolved a high capacity WM, this system is likely a general biological prerequisite to higher cognition.

## Materials and Methods

### Subjects

Two hand raised male carrion crows (*Corvus corone*) of 1 years of age, with baseline weights of 508 and 500 g, were used. The animals were group housed in a spacious aviary at 12 hours day/night cycle. During training and behavioral testing the animals were on a food protocol such that food-pellets (BEO special, Vitakraft, Bremen, Germany) could be used as a reward during the experiments. Access to water and grit was unrestricted, the animal’s body weight was controlled daily. When not in experiments, the birds were given food *ad libitum*. All procedures were carried out in accordance with the National Institutes of Health *Guide for Care and Use of Laboratory Animals* and were approved by the local ethical committee and authorized by the national authority (*Regierungspräsidium*).

### Experimental setup

The setup consisted of a chamber (50 cm (width) × 50.5 cm (depth) × 77.5 cm (height)), equipped with a 22″ touchscreen (ELO 2200 L APR, Elo Touch Solutions Inc, USA), a perch placed such that the distance from the bird’s eye to the screen was 8 cm, a camera (Sygonix, Nürnberg, Germany) for remote monitoring and a custom-made automatic pellet feeder (plans available: www.jonasrose.net). The position of the animal’s head was tracked in the horizontal and vertical plane, additionally the rotational angle of the head was tracked in both dimensions. For head-tracking, two open-source computer-vision cameras ‘Pixy’ (CMUcam5, Charmed Labs, Texas, USA) were used. The cameras reported location and angle between two LEDs. LEDs were located in a lightweight 3d-printed mount that was attached to a lightweight surgically implanted head-post and removed after each experimental session. The system reported head-location at a frame rate of 50 Hz and data was smoothed by integrating over 2 frames in Matlab using custom programs on a control PC. All experiments were controlled by custom programs in Matlab using the Biopsychology^56^ and Psychophysics toolboxes^57^. Digital input and output of the control PC was handled by a microcontroller (ODROID C1, Hardkernel co. Ltd, Anyang, South Korea) running custom software (available: www.jonasrose.net) connected through gigabit network.

### Behavioral protocol

Each bird was tested daily. During experimental sessions drinking water was periodically offered to the animals. The crows were trained to perform a change localization task^58^ that was carefully modeled on a design recently used with primates^29^. The paradigm allowed to test performance under varying WM-load (Fig. 1A). Following an inter-trial interval (2 s), a red dot was presented in the center of the screen (maximal 40 s). Animals initiated the trial by holding the head centered in front of the stimulus for 160 ms. This caused the stimulus to disappear and an array of two to five colored squares to appear 100 ms later. During this stimulus-presentation (0.8 s) the animals had to hold the head still (less then ± 2 cm horizontal or vertical displacement) and look straight at the center of the screen (less than ± 20° horizontal or vertical rotation) or the trial was aborted. Following this stimulus-period, the array disappeared for a short memory delay (1 s) after which the stimulus-array reappeared with one color exchanged. In order to obtain a reward, the animal had to indicate which color was exchanged with a single peck to that stimulus. An incorrect response to one of the unchanged colors or failure to respond (within 4 s), was mildly punished by briefly flashing the screen white and with a time-out (10 s). As reward, food pellets (BEO special) were delivered to a receptacle on the feeder which was illuminated during reward-delivery (2 s). Reward was delivered probabilistically to increase the trial-number such that 55% of correct responses result in primary (food) and secondary (feeder illumination) reinforcement, on the remaining trials only secondary reinforcement was delivered.

Design of the stimuli was based on the protocol by Buschman *et al*.^29^. On each trial two to five colors were presented at fixed screen locations. For each day, random color-combinations were chosen from a set of 14 colors (two pairs excluded for similarity) such that six pairs, one for each stimulus-location were chosen on a given day. Thus, on each training day, one random pair of colors was fixed to each of the six stimulus locations. The order of presentation of the colors within a pair was randomized and balanced across trials. The target location (color-change), the total number of stimuli in the array and the number of stimuli on the target hemifield were randomized such that all conditions had equal likelihood on a given trial. Color-stimuli were square, 10 degrees of visual angle (DVA) on either side and placed either on the horizontal meridian of the screen or 45.8 DVA above/ below the meridian at a distance from the center of 54 and 55.4 DVA (center of the stimulus) respectively (Fig. 1A). The maximal binocular overlap for carrion crows is around 37.6 DVA^59^; thus all color-stimuli were placed well outside the binocular area, taking into account head-movement, head-rotation and eye movement (Fig. 1B).

### Data analysis

All data analysis was performed in Matlab (Mathworks inc. Natick, Massachusetts, USA) using statistics and curve fitting toolboxes. In the literature, different methods to estimate WM-capacity are reported that can result in very different estimates^60^. Our analysis largely followed protocols described for monkeys in order to ensure comparability of the results^29, 46^. All analyses were based on attempted trials (with the exception of gaze breaks, based on started trials) where the animals responded either to a correct or an incorrect stimulus at the end of the trial.

The effects of number of stimuli on performance, gaze breaks and reaction time were assessed with analysis of variance (ANOVA) with number of stimuli as single, continuous factor. The effects of number of stimuli ipsilateral and contralateral to the target were assessed with a two-factor (continuous factors) ANOVA across trials (analysis was chosen for comparability with^29^). Differences in reaction time between correct and incorrect trials were assessed with a single factor ANOVA. In addition, the effect of number of stimuli ipsi- and contralateral to the target were tested with a general linear model with logistic link function and a binomial distribution as a more suitable analysis for binary data.

An information theoretical approach was used in order to estimate the information animals had about the stimulus display^29, 46^. Here, we computed the mutual information about the stimulus identity given animal’s choice (equation (1)).

1 $$I(R;S)=H(S)-H(S|R)={\sum }_{R}{\sum }_{S}P(r)P(s|r)\,lo{g}_{2}\,(\frac{P(s|r)}{P(s)})$$

where $P(r)$ is the probability of response to a particular location, $P(s)$ is the probability of the target occuring at each location, $P(s|r)$ is the conditional probability of target being at certain location given that animal chose that location.

The most common measures of capacity in the literature, Pashler’s and Cowan’s K, could not be used in the present study since animals were to *localize* a change and not report *if* an item changed. Hence, we used an estimate of capacity (K) that was previously applied to primate data and also used in comparable paradigms with humans^46, 47, 61^. Capacity (K) was estimated for each WM-load by: $K=n\ast p$, with *p* being proportion correct and *n* the number of items in WM. Additionally, we used a modelling approach to estimate capacity reported for the primate data (equation (2)).

2 $$p(n)=\,\{\begin{array}{c}b,\,n\le c\\ b\frac{c}{n},\,n > c\end{array}$$

This approach assumes that the animals perform at baseline-rate (*b*) until the number of items in display (*n*) reaches capacity (*c*) at which point performance decreases.

### Surgery

Both animals were chronically implanted with a light-weight head-post to attach a small LED-holder during behavioral experiments. Before surgery, animals were deeply anaesthetized with ketamine (50 mg/kg) and xylazine (5 mg/kg). Once deeply anaesthetized, animals were placed in a stereotaxic frame, the feathers were removed over the base of the beak and a small incision was made to retract the skin. A small opening was drilled in the surface of the bone in order to expose the *trabeculae* to which the head-post was attached with dental acrylic. The wound-margins were sutured and following the administration of analgesia (morphasol, 1.5 ml/kg) the animal was placed in a recovery-cage.

### Data availability

The datasets generated during and/or analyzed during the current study are available from the corresponding author on reasonable request.

## Electronic supplementary material

Supplementary Figures
