# Supplementary material for: Crows Rival Monkeys in Cognitive Capacity
Source: Sci Rep. 2017 Aug 18;7:8809. doi: 10.1038/s41598-017-09400-0 (PMC5562807; doi:10.1038/s41598-017-09400-0)
Supplement: Supplementary file 1 — Supplementary Figures [file 41598_2017_9400_MOESM1_ESM.pdf]

Crows Rival Monkeys in Cognitive Capacity

Dmitry Balakhonov, Jonas Rose*

Dept. Of Psychology, Ruhr-University Bochum, 44801 Bochum, Germany.

E-mail (corresponding author): *jonas.rose@ruhr-uni-bochum.de*

Supplementary figures

Figure S1

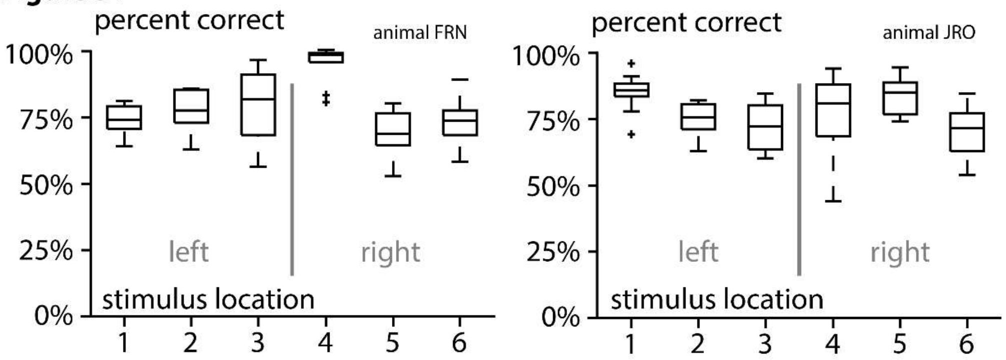

Fig. S1. Performance of each crow on attempted trials as a function stimulus-location. Locations 1, 2 and 3 are visible to the left eye, locations 4, 5 and 6 are visible to the right eye. While there are slight differences in performance on the different stimulus locations (especially crow FRN, location 4), both animals perform at a high level on all locations and with both eyes.

Figure S2

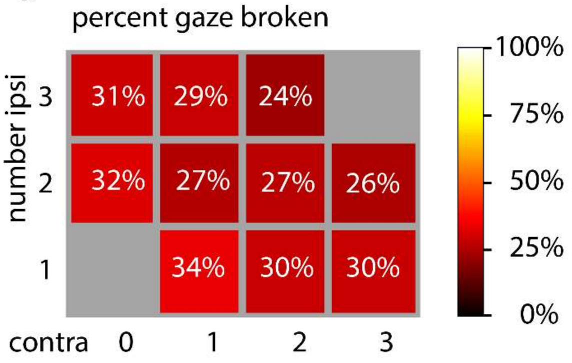

Fig. S2. Gaze-breaks are not modulated by number of items ipsilateral/ contralateral to the target. Gaze-breaks of both animals as a function of number of stimuli on target side (number ipsi) and number of items contralateral to the target (number contra). Background shading indicates percent correct on attempted trials.
